# Individual Differences in Learning and Cessation Predict Academic Performance

**DOI:** 10.3390/bs16081436

**Published:** 2026-08-20

**Authors:** Ashley L. Macchione, Jeffrey Coldren

**Affiliations:** Department of Psychological Sciences and Counseling, Youngstown State University, Youngstown, OH 44555, USA; alytle03@student.ysu.edu

**Keywords:** learning cessation, self-regulation, error rate, individual differences, academic performance

## Abstract

Most research on learning emphasizes knowledge learning, yet an equally important skill is knowing when to stop. This study examined learning cessation—when to disengage from further attempts once performance has stabilized—and its relation to academic performance. A sample of 183 undergraduates completed three perceptual discrimination problems varying in solvability and mastery criteria. Performance curves were analyzed by measuring error rates, allowing for the measurement of performance within the learning and cessation phases. Learners demonstrated wide variability across all three problem types, persisting well beyond the point at which continued attempts offered additional value. Individual differences in cessation behavior were associated with academic outcomes, though the pattern varied by problem type and outcome measure. Efficient cessation in a solvable task predicted higher ACT scores and high school GPA, while more attempts on the unsolvable problem showed a positive association with ACT performance and university GPA. Findings highlight the educational relevance of regulation efficiency, suggesting that the ability to regulate not only how long to attempt learning but also when to cease is a critical dimension of effective learning.

## 1. Introduction

While most investigations of learning focus on acquiring knowledge, few address its equally vital counterpart: knowing when to stop. It is the thesis of this paper that to be effective, a learner must regulate between two complementary states: learning and cessation (Shultz et al., 2012). Learning is the deliberate intake of information guided by feedback from the environment (Shteingart & Loewenstein, 2014; Subramanian et al., 2022) through which knowledge and skills are amassed to solve problems or reach a level of mastery. Cessation, by contrast, involves halting further attempts at learning. Once sufficient information has been gathered and no additional effort improves performance, learners risk wasting time, energy, and limited cognitive resources on fruitless attempts. Autonomous learners must strike the optimal balance to gather enough information to reach a solution but stop before scarce time, energy, and limited cognitive resources are wasted through unproductive learning attempts (Bazerman & Moore, 2009; Lucas et al., 2015; Murayama et al., 2016) and fail to take advantage of more fruitful learning opportunities (Buchanan, 1991). The need for cessation is readily apparent under learner-controlled, rather than experimenter-controlled conditions, as most learning occurs under naturalistic conditions in which the outcome is open-ended and unspecified. Therefore, the learner must decide when a satisfactory level of performance has been attained.

A previous investigation explored this thesis by asking how quickly and under what conditions college students would cease making attempts at learning (Coldren, 2023). The task involved having participants solve three perceptual discrimination problems that varied by whether they were solvable, capable of being mastered, or could never be solved. Participants were given the explicit choice on every trial whether to continue or end the problem. Thereby, the task assessed cessation under in situ conditions in real-time as the problem unfolds, and under the control of the learner. It was expected that, with nothing to gain from continued learning after mastering a problem or realizing the problem was unsolvable, participants would end attempts quickly. The results ran counter to this expectation. When given the explicit option to quit making attempts to solve problems, many learners did not quit even though they had attained nearly perfect performance or reached a point of futility.

These findings do not fit within any of the extant theoretical perspectives that address the strategic stopping of behavior, as reviewed below.

### 1.1. Theoretical Perspectives

#### 1.1.1. Self-Regulated Learning Model

Learning cessation may be considered a component of the broader self-regulated learning (SRL) framework, which involves setting goals, monitoring progress toward those goals, and modifying behavior accordingly (Butler & Winne, 1995; Winne, 2001). A substantial body of research demonstrates that proficiency in these processes predicts improved academic and educational functioning (Dent & Koenka, 2015; Pintrich & DeGroot, 1990; Theobald, 2021). From this perspective, the SLR model generally includes the ability to terminate ineffective attempts as supporting efficient learning but it does not specify the actual conditions under which learners do stop learning (Eskreis-Winkler & Fishbach, 2019), nor does it account for cases in which learning continues beyond what is effective to solve the problem (Lucas et al., 2015). This may be due to the reliance of the SLR model upon self-report questionnaires that may not reflect actual learning performance in natural learning situations (Friedman & Gustavson, 2022; Ventura et al., 2013; Zhou & Winne, 2012).

#### 1.1.2. Meta-Cognitive Control

A central theme in cognitive psychology is that learners regulate their study time through metacognitive monitoring of learning progress, with the decision to cease studying reflecting an ongoing judgment of whether sufficient learning has occurred. The discrepancy reduction model proposes that learners allocate effort toward reducing the gap between current knowledge and a target learning criterion (Dunlosky & Hertzog, 1998; Dunlosky et al., 2013; Nelson & Dunlosky, 1991). This model, however, struggles to account for the labor-in-vain effect, whereby learners invest disproportionate time in difficult items despite minimal gains (Nelson & Leonesio, 1988). Subsequent theoretical refinements have sought to resolve this tension. The diminishing criterion model suggests that learners lower their internal performance goals over time when facing persistent difficulty (Ackerman, 2014; Undorf & Ackerman, 2017), while the Region of Proximal Learning framework proposes that learners preferentially study material just beyond their current mastery and use perceived learning rate to stop making attempts; learners continue to study when progress feels rapid and disengage when it appears to plateau (Metcalfe, 2002, 2009; Metcalfe & Kornell, 2003, 2005).

Empirical evidence indicates that such metacognitive stopping decisions may be suboptimal, and that errors occur in both directions. Some learners may quit prematurely. For example, Murayama et al. (2016) found that college students allowed to self-terminate study of a text often stopped too early and subsequently recalled less material than peers who continued studying, while Krogulska et al. (2021) showed that adults faced with an expanding word list tended to stop early to avoid subjective overload. This decision paradoxically reduced recall, with participants largely unaware that early stopping carried this cost. Conversely, other findings suggest learners can appropriately adjust studying to task solvability. Payne and Duggan (2011) demonstrated that students abandoned unsolvable problems more quickly than solvable ones, consistent with a rational allocation of effort toward attainable goals. More recently, Law et al. (2022) identified a “giving-up” factor linked to opt-out behavior on cognitive tasks that was associated with higher academic performance, suggesting that individuals who more frequently opt out of answering as an adaptive strategy tend to have higher academic achievements. Collectively, this perspective underscores that learners may disengage too early due to misjudged potential gains, or persist too long due to overestimated likelihood of success, revealing errors in metacognitive self-regulation.

#### 1.1.3. Executive Functioning

Inhibition constitutes a key component of the executive functioning perspective, a broader set of control processes governed largely by the prefrontal cortex and responsible for monitoring and regulating goal-directed behavior (Miyake et al., 2000). Specifically, inhibition refers to the capacity to suppress nonproductive or prepotent responses, a mechanism that may extend beyond simple response competition to the domain of self-regulated learning. Appropriately disengaging from an ineffective or unproductive study episode may itself represent an act of inhibitory control, requiring the learner to override the prepotent tendency to persist with an established behavior (van der Ven et al., 2013). Viewed this way, the decision to cease studying is not purely a metacognitive judgment of enough learning but may also depend on the inhibitory resources needed to act on that judgment, suggesting that learners with weaker inhibitory control could struggle to stop studying even when they recognize further effort is unproductive. Executive control processes have been linked to academic and intellectual performance during the elementary school years (Spiegel et al., 2021; St Clair-Thompson & Gathercole, 2006), although their relationship to performance in adulthood remains comparatively less established.

Recent work provides some support for this relationship among adults. Dvorak (2024) found a weak but significant negative correlation between the number of color identification errors made during the Stroop Test and grade averages, indicating that students with lower grades tended to make more mistakes. Coulanges et al. (2021) reported a significant positive relationship between the number of correct responses on Stroop performance and academic performance, indicating that individuals with higher Stroop task scores tend to achieve higher scores on math achievement tests among university students. Both studies find that adults who perform the Stroop task more accurately tend to have better academic outcomes; they differ mainly in whether that accuracy is interpreted as inhibitory control or as conscientiousness/task engagement, and in whether the outcome is broad GPA or a math-specific measure. Further, while important theoretical distinctions exist between inhibition as indexed by the Stroop task and behavioral measures of learning cessation, these findings suggest that individual variation in the capacity to disengage from a task is associated with academic performance and raise the possibility that inhibitory control may serve as a common mechanism underlying individual differences in when and how effectively learners choose to stop studying, although this is untested.

#### 1.1.4. Predictive Processing Theory

A utilitarian account of learning cessation emerges from predictive processing theory, which frames learning as a fundamentally value-driven process aimed at optimizing behavior while minimizing metabolic and cognitive cost (Friston, 2010). Within this framework, learners continuously refine internal generative models by comparing top-down, representation-based predictions against incoming sensory or task-based observations, updating their models in proportion to the resulting prediction error (Koster et al., 2020; Sprevak & Smith, 2023). Learning is thus not governed by an explicit judgment of enough knowledge but instead unfolds as a self-terminating process. As internal models become increasingly accurate and prediction errors diminish, the impetus for further updating recedes, and learning ceases once errors approach a minimum (Friston, 2010). This account offers a complement to the metacognitive and inhibitory perspectives on learning cessation discussed above. Whereas discrepancy-based and region-of-proximal-learning models emphasize a perceived sense of learning progress as the basis for stopping (Metcalfe & Kornell, 2005), predictive processing suggests that learning rate reflects underlying model accuracy rather than an independent metacognitive judgment.

This reasoning has been implemented computationally in supervised neural network models, in which internal states are iteratively updated against target values until the error rate between predicted and correct outputs stabilizes at a low level, signaling that accurate learning has been achieved. Shultz et al. (2012) examined this process directly, training a supervised neural network to classify perceptual stimuli into one of four categories and indexing learning progress by the degree of error reduction between the model’s classification accuracy and correct target values. Simulations showed that the model continued adjusting its parameters as long as error was decreasing, ceasing only once error reached a stable, low asymptote.

#### 1.1.5. Limitations of Existing Theories for Explaining Learning Cessation

The finding that many learners continued making attempts long after attaining near-perfect performance or reaching a point of demonstrable futility suggests that existing theoretical perspectives, while each capturing important components of learning cessation, may not fully account for the specific temporal dynamics of when and why learners choose to stop. The self-regulation learning framework provides a valuable overarching structure by identifying termination as a component of efficient self-regulation, though it has not yet been extended to specify the precise conditions under which stopping occurs in real time, particularly in cases where responding continues beyond apparent goal attainment (Eskreis-Winkler & Fishbach, 2019; Lucas et al., 2015). Similarly, metacognitive models such as discrepancy reduction, diminishing criterion, and Region of Proximal Learning offer compelling accounts of how a perceived judgement of goal attainment can prompt disengagement (Metcalfe & Kornell, 2005). Executive-functioning accounts likewise offer a promising yet untested mechanism about the inhibitory resources needed to disengage from an ongoing task. Predictive processing theory, for its part, offers a convincing formal account of how prediction error should approach a minimum as learning concludes (Friston, 2010); building on this work, it remains an open question whether overt behavioral cessation tracks this internal accuracy directly.

Taken together, these perspectives share the assumption that learning cessation reflects a response to some signal of diminishing returns, whether that signal is framed as a felt discrepancy from a goal, an inhibitory override of a prepotent behavior, or a decline in prediction error. None, however, can adequately explain why some learners persisted well beyond the point at which that signal should have prompted disengagement. This gap exists in part because none of the theoretical perspectives reviewed specify a concrete, trial-level marker of when a learner’s performance has effectively stopped improving, making it impossible to determine, for any individual learner, whether continued attempts reflect a failure of self-regulation, an insensitivity to error feedback, or a response to uncertainty about task solvability. Existing accounts have also largely relied on self-report, group-level, or offline outcome measures, none of which can capture cessation as it unfolds moment-to-moment within a single, learner-controlled task. As a result, there is a need for a method to empirically separate the learning phase, in which performance is still changing, from the cessation phase, in which it has stabilized, and for quantifying how many attempts are made once that stabilization point is reached.

The present study addresses this need directly by adapting an error-stabilization criterion from machine learning to identify, on a trial-by-trial basis, the point at which each learner’s performance curve stabilizes, thereby operationalizing the boundary between learning and cessation. This approach allows us to move beyond asking whether learners eventually stop and instead ask precisely when they stop relative to their own demonstrated learning, and whether the resulting variability in learning and cessation behavior helps explain extended learning attempts and its relation to broader academic outcomes.

### 1.2. Research Questions

#### 1.2.1. RQ 1: Can Learning Cessation Results Be Replicated?

Given the novelty of the learner-controlled task using problems that vary in solvability, and the finding of extended learning attempts for problems that offer little apparent progress, the essential findings of Coldren (2023) should be replicated to move forward with an extended analysis of the learning and cessation phases.

#### 1.2.2. RQ 2: Do Human Learners Use Error Rate to Regulate Learning?

To test that humans regulate their learning in relation to their progress, cessation must be dissociated from learning to isolate each one’s unique variability. Group-based performance, summary statistics, and observed decisions such as whether to quit or continue a learning task are not sufficient to reveal the performance, processes, and variability of individual learners (Robinson, 2011; Stern, 2017). While there have been some attempts to define distinct phases of the learning curve, none have explicitly identified the cessation phase per se (Fitts & Posner, 1967; Tenison & Anderson, 2016). Doing this involves finding the point at which learning progress stabilizes and using that point to dissociate the learning phase from the cessation phase. We may then explore the extent of variability in each phase, and their influences upon concomitant measures.

#### 1.2.3. RQ 3: Does Variability in Learning and Cessation Have Implications for Education?

The ability to cease making attempts during learning bears directly on education’s broader goals of fostering lifelong and generalizable learning skills. Contemporary educational theory increasingly favors learner-centered approaches over instructor-driven instruction (Herrington et al., 2014; Scheiter, 2020), placing greater responsibility on students to regulate their own learning processes, including determining when continued effort is productive and when discontinuing attempts is optimal (Allen et al., 2011; Bernardo et al., 2019; Kokotsaki et al., 2016; Wood, 2003). This places substantial weight on learners’ self-regulatory capacities, although Atkinson (1972) raised doubts about whether learners can reliably make such judgments without more instructor-directed guidance. This same need for learning self-regulation is reflected in a utility-based view of education, wherein students seek to maximize achievement given limited resources such as time and cognitive effort (Walberg, 1982) and must therefore allocate effort toward tasks offering the greatest academic return, drawing on skills such as time management (Kitsantas et al., 2008; Stolzenberg et al., 2020) and accurate awareness of one’s academic strengths and limitations (Sahranavard et al., 2018) to implicitly judge when continued attempts at a task are no longer efficient. Taken together, these educational perspectives underscore that the decision to cease making learning attempts may vary across learners and contexts, reinforcing the need to examine variability in both the learning and cessation phases as a means of understanding why some students stop appropriately while others persist well beyond the point of productive return.

## 2. Materials and Methods

### 2.1. Participants

Undergraduate students from the departmental participant pool volunteered for this study. The university IRB approved the project. Participants signed a consent form that assured their anonymity, stated their right to withdraw, and explained the option to choose an alternative assignment to complete the research requirement.

The final sample included 183 undergraduate students: 32% identified as female and 25% identified as non-Caucasian. The average age of participants was 20.23 years (*SD* = 4.25) and the mean credit hours completed was 33.71 (*SD* = 27.48). Participants also answered questions about measures of academic performance, including their university GPA at the time of the study (*M* = 3.29, *SD* = 0.55), their GPA upon graduation from high school (*M* = 3.49, *SD* = 0.47), and their ACT score (*M* = 22.74, *SD* = 4.09).

### 2.2. Procedure

Participants solved three consecutive perceptual discrimination problems that varied in problem solvability and solution criteria (e.g., Coldren, 2023). Each problem consisted of a pair of abstract geometric figures with four co-varying perceptual dimensions. The features were counterbalanced across blocks of eight trials so that all attributes appeared in every position. This was an inductive feedback-based learning task in which the correct choice was initially unknown to the participant; they were to select the one feature that was consistently rewarded over trials. Participants pressed the left or right keyboard arrow keys to indicate their choice. Feedback about the correctness of the choice (correct or not correct) appeared on the screen after every response. Participants were given verbal (at the beginning of every problem) and written (at the bottom of every trial screen) instructions informing them of the option to quit the current problem.

Participants gained familiarization with the procedure through two practice problems, which were followed by the three experimental problems. A perceptual discrimination task was used because problem solvability could be precisely controlled by the experimenter. Each experimental problem varied by whether a solution was possible and whether an external criterion for success was imposed upon the learner. These problem types were developed for this study. Participants were instructed to attain as many correct responses as possible in a row until they felt confident that they had solved the problem. The maximum trials a participant could attempt was 64.

The first problem was solvable, meaning that the feature designated as correct was to be selected by the learner. Further, an external learning criterion defined as eight consecutive correct responses was imposed upon the learner. This problem served as an analog to typical mastery-learning conditions.

The second problem also contained a correct feature but did not include a criterion for problem solution. Without any constraint to end learning, participants could make as many attempts as they wished until reaching the maximum of 64 trials. As a result, participants could attain a high level of success if they did not end the problem. This was called the overlearning problem.

The third problem was unsolvable as no individual feature was consistently rewarded and only 50% of the trials contained any reinforcement regardless of the response. This problem also did not contain a learning criterion so the participant could continue unabated until reaching the maximum of 64 trials. It was expected that learners would likely attain a moderate and constant error rate as the level of performance hovered around chance levels over trials (e.g., 50%).

Measures from the task included the number of trials attempted on each problem and the outcome of each problem (i.e., whether the participant solved the problem, quit the problem, or attempted the maximum number of trials).

## 3. Results

Analyses are organized by the research question posed in the introduction. All data and analyses are available at Coldren (2026).

### 3.1. RQ 1: Can Learning Cessation Results Be Replicated?

**Figure 1:**
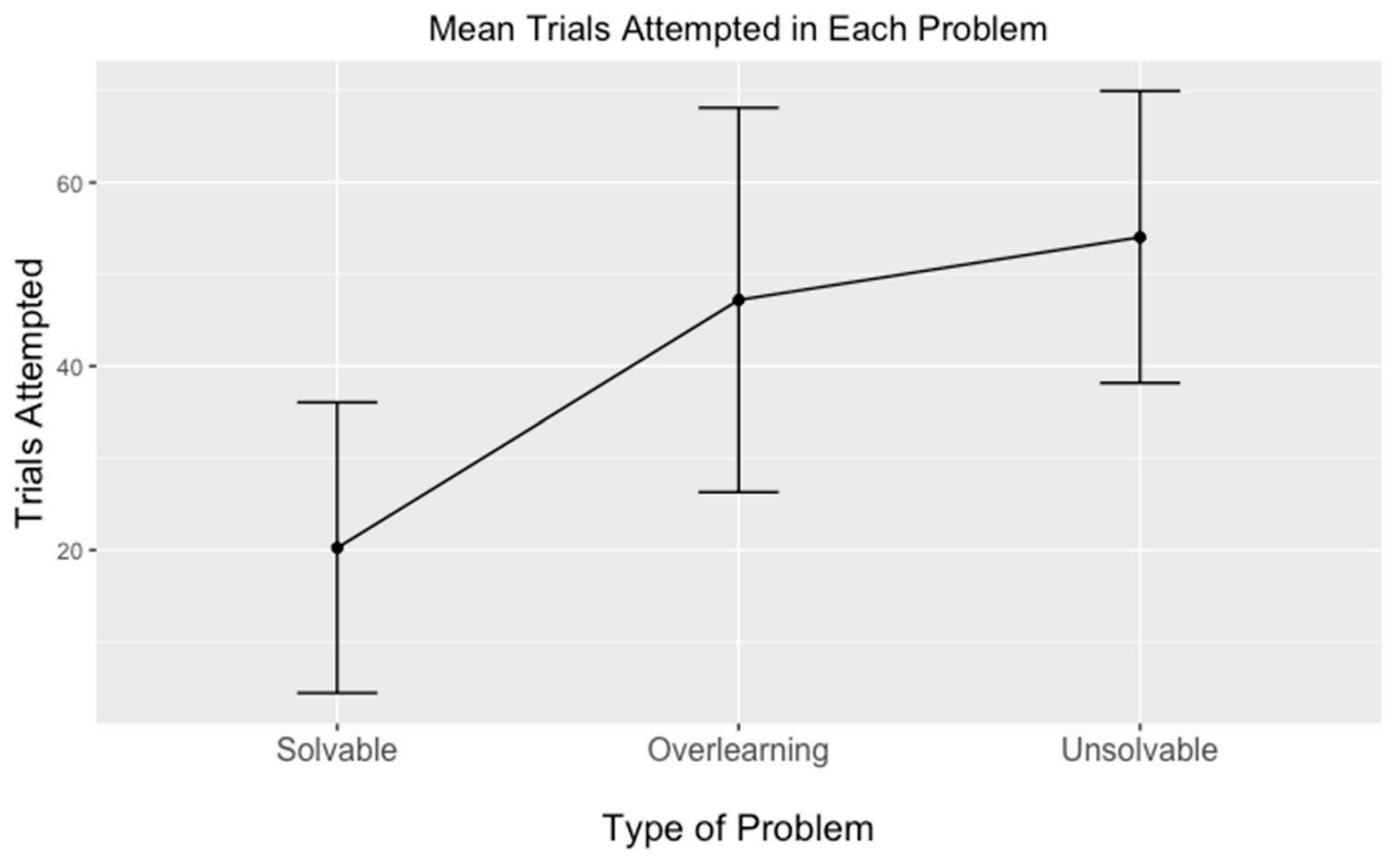
Mean Number of Trials for Each Problem (Error Bars Represent *SD*).

The number of attempted trials increased across the solvable, overlearning, and unsolvable problems, as revealed in a one-way related-sample ANOVA (*F*(2, 364) = 285.4, *p* < 0.05) (See Figure 1). All Bonferroni pair-wise comparisons were significant. Thus, learners found the overlearning and unsolvable problems to be more challenging than the solvable problem.

**Figure 2:**
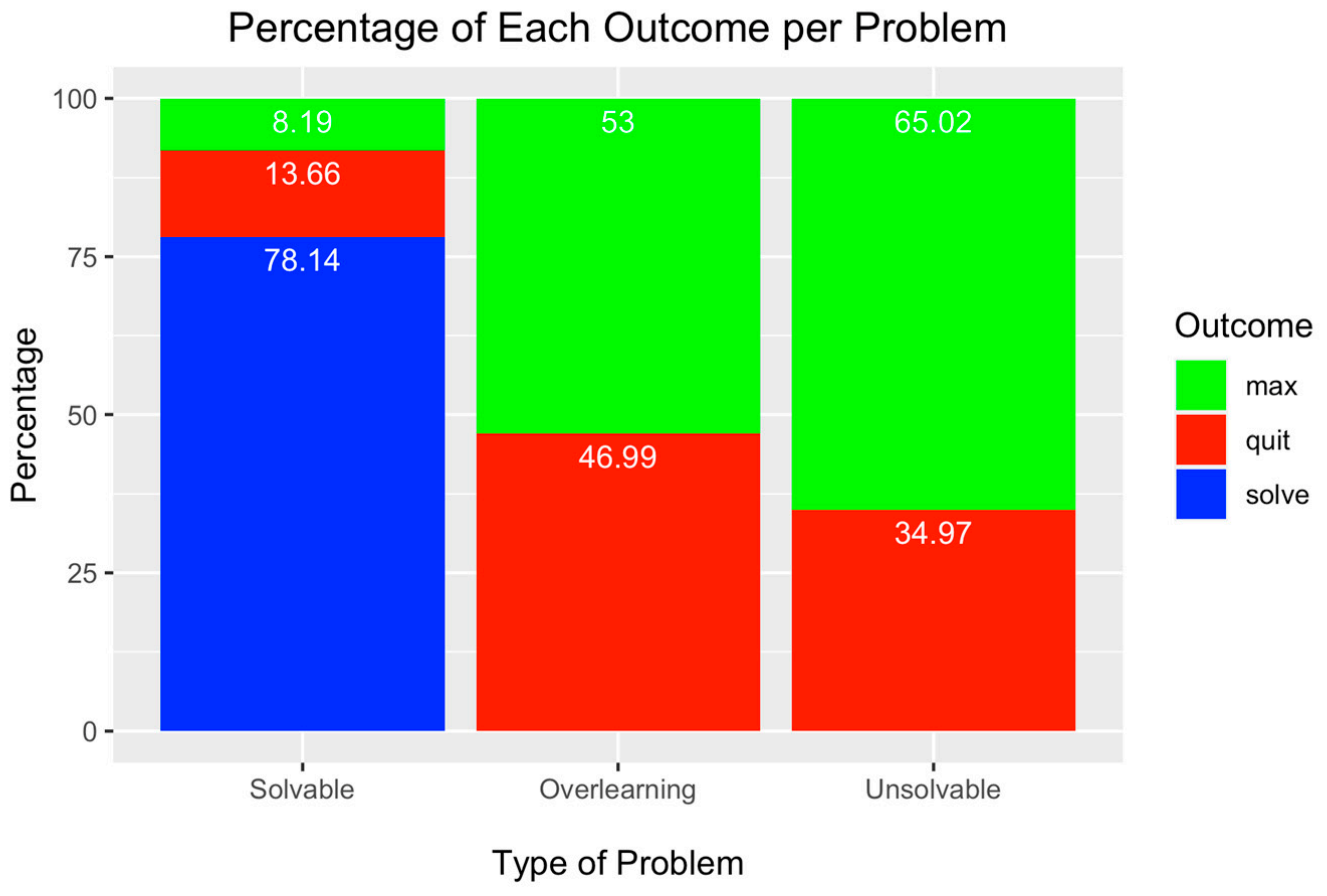
Percentage of Outcomes for Each Problem.

Second, participants’ solutions for each problem were examined. One of three outcomes was possible: solve the problem, quit the problem, or continue until reaching the maximum number of trials (e.g., 64). It was not possible to solve the overlearning or unsolvable problems. The percentages for each outcome for the problems are shown in Figure 2. The majority solved the first problem, whereas only a few quit or reached the maximum number of trials. These percentages were significantly different from chance level performance of 33% (χ^2^(2, N = 183) = 90.8, *p* < 0.05). There was also a significant difference in outcomes for the unsolvable problem (χ^2^(1, N = 183) = 9.03, *p* < 0.05), with most participants reaching the maximum number of trials rather than quitting attempts. There was no significant difference between the problem outcomes for the overlearning problem (χ^2^(1, N = 183) = 0.36, *p* = 0.55). This pattern of results replicates those reported in Experiment 2 of Coldren (2023) and establishes the robustness of the phenomenon to justify later analyses.

### 3.2. RQ 2: Do Human Learners Use Error Rate to Regulate Learning?

The critical question is how to quantify and isolate variability in cessation as separate from learning. The measures just analyzed, the number of attempted trials and problem outcome, are not suitable to identify individual differences in cessation for the following reasons. First, the total number of attempted trials does not provide information regarding the correctness of participant answers. For example, two participants could have attempted the same number of trials, but one participant could have reached that point by answering all the questions correctly, whereas the other could have only answered half of the questions correctly. Further, the number of attempted trials does not provide information about the outcome of the problem. A participant who attempted 64 trials could have quit on the last trial or could have attempted the maximum number of trials. Second, the solution to the problem does not give any indication of when cessation occurred. If a participant quit a problem, it would not be possible to distinguish whether they quit on the 2nd trial or the 63rd trial. Further, examining only problem outcome would not reveal anything about the correctness of participant answers. For example, if a participant attempted the maximum number of trials in the overlearning problem, they could have always answered correctly, always answered incorrectly, or could have had some mix of responses.

#### 3.2.1. Examination of Error Rate to Determine Stability

Given the deficiencies of using attempted trials or problem outcome for identifying individual differences, we adopted an approach from machine learning to examine error rate (Shultz et al., 2012) to examine how well it could characterize human learning. Specifically, error rate in a neural net model is the difference between the accuracy of the current responses and their target (i.e., correct) values. Early in the training period, the error rate typically falls precipitously, but as learned responses become closer to their target values, the error rate eventually stabilizes at a low level and the model stops learning.

**Figure 3:**
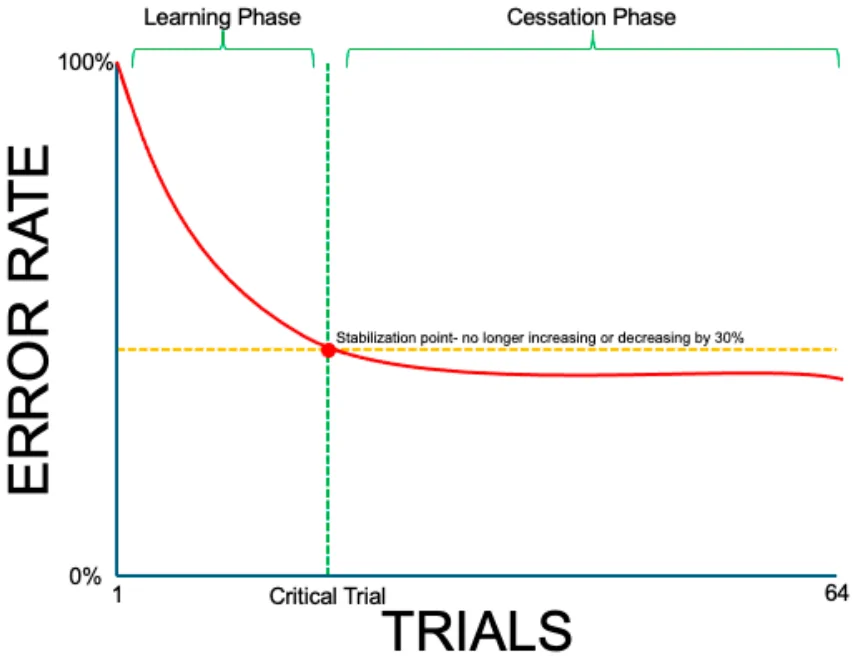
Learning Curve Anatomy.

With this model as guidance, we calculated cumulative curves for each participant’s incorrect responses over trials. We then identified the point at which the error rate for each participant stabilized (CritTrial), defined as when error rate did not change more than 0.30 from the previous trial. During learning as responses are becoming more accurate and aligned toward their final target outcomes, it was expected that changes in the error rate would be greater than 0.30 from the previous trial. Once the error rate stops changing by more than 0.30 from the previous trial, stabilization occurs and marks the demarcation between the learning and cessation phases. However, the error rate could be reset to a higher criterion if there was an upward or downward change in the accuracy of responding (Horowitz et al., 1972). Further, the change in error is relative to the previous trial, not from the beginning of the session. Thus, as error rate drops, the range for error becomes more narrow, reflecting stabilization. Figure 3 provides a hypothetical visualization for the phases of a learning curve.

**Figure 4:**
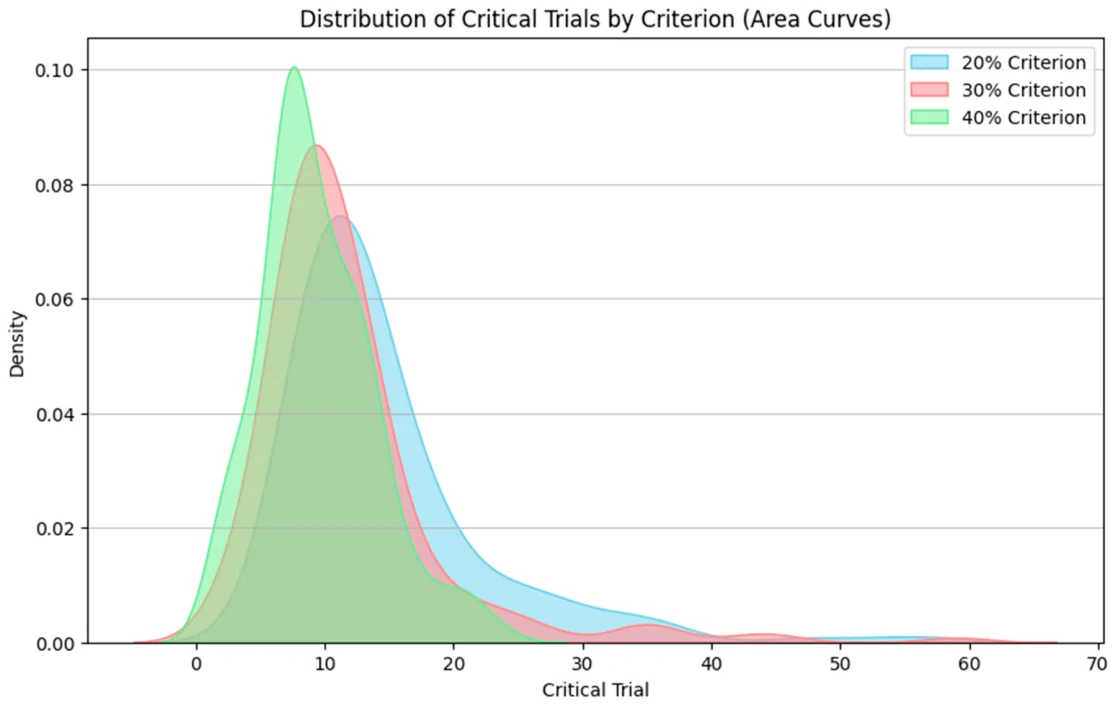
Comparison of Change Criteria.

Some explanation of the choice of 0.30 as the criterion for defining change in error rate is necessary as there is no precedent for this criterion in the literature on human learning that we are aware of. Ours was a novel investigation of this phenomenon, and therefore, we had to choose a model parameter. This is not unique to the present case because in any definition of curve stabilization, a value is required that specifies the range indicating when changes cease, thereby marking a period of stability. This applies whether one is using a behavioral criterion measure (as in the present case) or a mathematically defined derivative specified with epsilon (ε). If the change criterion is set too low, and therefore too stringent, stabilization may occur later due to the presence of small trial-to-trial fluctuations. Conversely, if the change criterion is set too high, and thereby more liberal, the curve may show early stabilization, as large variations can fall within the stabilization range. We tested these scenarios by calculating curve stabilization under three different change criteria (0.20, 0.30, and 0.40) for the solvable problem. As shown in Figure 4, the results were as predicted: The 0.40 change criterion yielded earlier stabilization points (e.g., CritTrial), whereas the 0.20 change criterion was the most conservative and produced stabilization points that were considerably later. Using a 0.30 change in error rate from the previous trial therefore represents a moderate and defensible choice.

#### 3.2.2. Identification of Phases of the Learning Curve

CritTrial was defined as the trial indicating the point of stability in error rate and thus indicates the number of trials comprising the learning phase; therefore, it was used to demarcate the transition between phases. TrialsToEnd was defined as the difference between CritTrial and the last trial attempted, and thus represents the number of trials in the cessation phase. Table 1 presents the descriptive statistics for each problem by phase.

**Figure 5:**
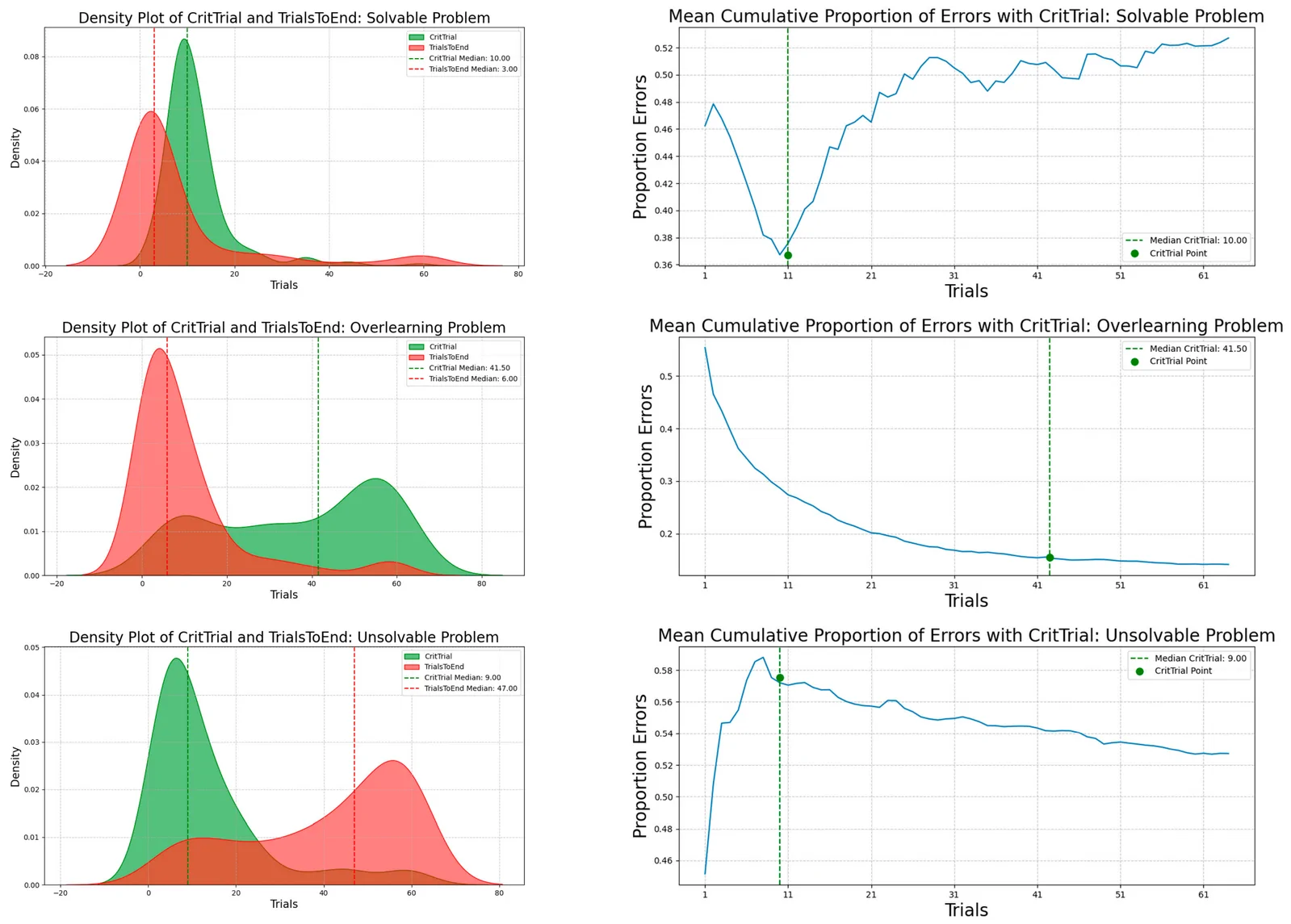
Dissociating the Learning and Cessation Phases.

The learning and cessation phases are graphically depicted in Figure 5. The left panels display frequency density plots of the trial on which participants attained the CritTrial and TrialsToEnd, respectively, and the right panels display the cumulative error curve over trials.

For the solvable problem, the error rate decreased rapidly over the first several trials before rising again, most likely reflecting the contributions of participants who did not ultimately meet the criterion for solving the problem. The dissociation between the learning and cessation phases is visible in the left panel: participants required a median of 10 trials to meet the learning criterion, yet tended to end their attempts relatively quickly thereafter, with a median TrialsToEnd of only 3 trials. Considerable variability is also evident in the right-hand tails of both distributions, reflecting the subset of learners who struggled either to meet the learning criterion, to end their attempts, or both.

The dissociation between the learning and cessation phases is more pronounced for the overlearning problem. Recall that this problem is structurally identical to the solvable problem, except that it contains no criterion for solution; learners could therefore continue unconstrained until they either chose to cease attempts or reached the maximum number of trials. As expected, the error rate declined steadily and eventually reached a very low level as performance became highly accurate. For the cessation phase, the median TrialsToEnd was only 6 trials, indicating that most learners stopped relatively quickly, yet the distribution exhibits a pronounced right-hand tail, indicating that a substantial proportion of learners persisted for an extended number of trials before ceasing attempts. This suggests that even when the correct stimulus feature had been identified, many learners did not terminate their attempts accordingly. Equally notable is the pattern in the learning phase: the median CritTrial was approximately 41.5, meaning that most learners required over 40 trials before performance stabilized. This is a surprising finding, because classical hypothesis-testing theory holds that once a learner selects the correct stimulus, they do not reject it (Levine, 1975). In this unconstrained, criterion-free context, however, learners appeared to resample and reselect among stimulus features, producing an extended learning phase. Taken together, the patterns in both the learning and cessation phases suggest that learners did not engage in the most efficient self-regulation of learning: they continued making attempts well beyond the point at which the error rate had stabilized.

The opposite pattern was observed for the unsolvable problem. In this condition, there was no consistently correct response; the reinforced stimulus feature varied randomly across trials. Consequently, errors were expected to stabilize around 0.50, that is, at chance-level responding. The error curve shows that the proportion of errors rose rapidly over the first several trials before gradually declining toward approximately 0.52, approaching but not fully reaching the expected chance baseline. The density plot further reveals that most learners reached the point of stabilization early in the learning phase, with a median CritTrial of only 9 trials, yet the majority persisted considerably longer, with a median TrialsToEnd of 47 trials. Thus, learners continued to make attempts despite the absence of any systematic change in error rate, suggesting a failure to recognize or respond to the lack of progress in this unsolvable condition.

### 3.3. RQ 3: Does Variability in Learning and Cessation Have Implications for Education?

Given the variability in the performance in each phase, the next analysis tested whether the number of trials during learning and cessation phases were related to academic performance.

Linear regression analyses were conducted on the educational outcome variables of ACT, university GPA, and high school GPA. For each outcome, the number of trials during each problem type (solvable, overlearning, and unsolvable) were analyzed by learning phase (learning or cessation). The statistical assumptions of linearity, homoscedasticity, and multicollinearity were examined for each model with no consequential departures noted. The results of the regression equations for all outcomes are shown in Table 2.

In predicting ACT, the overall model was significant (*F*(6, 143) = 3.60, *p* = 0.0024, *R*^2^ = 0.132). The only significant predictor during the learning phase was for the overlearning problem, such that fewer trials during initial learning were associated with higher ACT. During the cessation phase, significant predictions were observed for the solvable problem and the unsolvable problem. Specifically, fewer trials during the cessation phase of the solvable problem predicted higher ACT, whereas more trials during the cessation phase of the unsolvable problem predicted higher ACT.

For university GPA, the overall model was not significant (*F*(6, 124) = 1.645, *p* = 0.140, *R*^2^ = 0.073), but there was a borderline significant finding that more trials during cessation of the unsolvable problem was associated with higher university GPA. This is in line with the previous finding that more attempts during cessation also predict higher ACT.

The model predicting high school GPA was borderline significant (*F*(6, 160) = 2.06, *p* = 0.061, *R*^2^ = 0.0717). Fewer trials during the learning phase and the cessation phase of the solvable problem predicted higher high school GPA. More attempted trials during the cessation phase of the overlearning problem were associated with increased high school GPA, although this was only borderline significant.

## 4. Discussion

This study confirms our previous finding that most college students continued to attempt to solve problems in which they have achieved either near perfect performance or reached a point of futility (Coldren, 2023). This pattern runs counter to utility-based interpretations such as machine learning (Shultz et al., 2012) and predictive processing theory (Koster et al., 2020; Sprevak & Smith, 2023) that the efficient solution is for learners to stop making attempts once the error rate falls to a stable point.

The present experiment sought to answer whether human learners use and are sensitive to cost–benefit analysis such as error rate and use it to regulate their learning. Our method involved constructing cumulative performance curves for participants as they attempted three perceptual learning problems. Inspired by the performance of machine learning models, we examined when the error rate stabilized. This point was thereby used to divide performance into the learning phase (when the rates of errors continued to decrease) and the cessation phase (when errors stabilized). We were then able to dissociate and examine the amount of variability within each phase.

### 4.1. Variability in Learning and Cessation Phases

The results revealed distinct patterns of learning and cessation across the three problem types, consistent with the hypothesized dissociation between these two phases. For the solvable problem, learners reached the learning criterion in a moderate number of trials yet ceased attempts relatively quickly thereafter, though considerable individual variability was evident in both phases. The overlearning problem revealed striking dissociation of the phases. Despite the absence of a solution criterion, most learners eventually achieved a very low error rate, yet required a surprisingly large number of trials, well beyond what classical hypothesis-testing theory would predict, before performance stabilized, and many continued making attempts even after stabilization had occurred. In the unsolvable problem, where no consistently correct response existed, error rates approached but did not fully reach chance-level responding, and learners persisted for a markedly greater number of trials relative to the point at which their performance had stabilized, suggesting an insensitivity to the absence of systematic feedback. Across all three conditions, learners demonstrated high variability in their regulation.

### 4.2. Relations to Academic Outcomes

The present results identify variability in phases that predict educational success. In general, college students who can effectively cease learning have better educational outcomes. The conclusion, however, is nuanced by individual differences in the learning and cessation phases that varied by the academic outcome measured and the type of problem.

Academic outcomes were differentially predicted by performance during both the learning and cessation phases, with cessation emerging as the more consistent predictor across problem types. For the solvable problem, fewer learning trials predicted higher high school GPA, and fewer cessation trials predicted both higher ACT scores and higher high school GPA, suggesting that efficient task completion and timely disengagement are jointly associated with broader academic performance. For the overlearning problem, fewer learning trials predicted higher ACT scores, while cessation trials showed only a borderline association with high school GPA. For the unsolvable problem, neither learning-phase nor cessation-phase trials predicted high school GPA, but greater persistence during cessation predicted higher ACT scores and showed a borderline association with university GPA, suggesting that sustained attempts under unresolvable conditions may reflect a meaningful individual difference relevant to higher-level academic performance. Whether this type of performance reflects the learner’s grit (Duckworth & Gross, 2014) cannot be determined in this experiment, but is worthy of further investigation. Across all three problem types, the prediction accuracy for university GPA was the lowest, while ACT scores and high school GPA showed the strongest and most consistent associations with both learning and cessation behavior.

Of the three measures of academic success, individual differences in cessation were most evident for ACT performance and high school grade point average. ACT score is generally regarded as a more objective and reliable measure to capture academic performance as it measures general intelligence, *g*, as well as the knowledge and skills of students not covered by *g*. ACT scores have been shown to have a high predictive validity when examining cumulative college grades over a four-year degree (Schmitt et al., 2009).

We included grade point average as another measure of scholastic success. Whereas individual differences in cessation did relate to high school grade point average, there were no such relations for university grade point average. GPA may be confounded by issues of subjectivity and unevenness across teachers, classes, and schools. A possible problem may be that grading standards vary by teachers’ judgment and school environments (Brookhart et al., 2016), such as when intentionally challenging classes yield lower grades due to the nature of the class (Sadler & Tai, 2007).

The reasons why high school grade point average is related to individual differences in cessation rather than university GPA is not evident but may be due to the different nature of the high school experience and expectations. It is plausible that success in high school may be focused on tangible and concrete learning tasks, whereas university GPA also may include intellectual, cognitive ability, and self-regulation in learning as well as course-taking tactics and motivational issues like class attendance (Lei et al., 2001). Another issue is that university GPA was assessed when most of the students were in their first year and had relatively less exposure to the rigors of university education.

### 4.3. Theoretical and Educational Implications

These results present a more complex and nuanced conclusion about the ability of human learners to use error rate in deciding when to stop making attempts to solve a problem. As in our previous study, many college-aged learners used the strategy of continuing to make attempts at learning until they reached the maximum number of trials. In some problems, no solution was possible, and in others, the problems were mastered to the point of perfection. Although such continuation may be inefficient from a utilitarian perspective of learning, we must caution that these results should not be interpreted that such persistence is ineffective. In fact, continuing to attempt challenging problems may be beneficial to academic success.

Given that there are several theoretical perspectives and constructs that involve the topic of strategic stopping as reviewed in the introduction, one may question how the term cessation fits into this landscape. Without direct comparison of the constructs, it is impossible to say whether there may be some convergence or theoretical overlap. This remains to be investigated in future studies through discriminant analyses. Cessation, however, has the benefit of being defined directly in terms of the learning curve performance rather than relying upon the measurement from rating scales or other indirect process. Cessation also is measured during an in situ, real-time, learner-controlled task that has more ecologically relevance than other measures.

In considering where cessation fits with other theoretical constructs, the question arises whether variability during learning and cessation is related to constructs that indicate a valence toward a learning goal, including persistence, engagement, and grit. In other words, does cessation reflect a separate dimension from persistence, or do they represent opposite ends of the same continuum? Are learners who are more persistent as indicated by grit, for example, less likely to quit a task? A full accounting of why and how learners engage in a task requires empirical examination of the learners’ goals, motivation, self-monitoring, maturity, and experience.

The further implication of these findings for education is that they lend partial support for Atkinson’s (1972) finding that some students may not effectively monitor their learning. As such, it points toward an important qualification of the self-regulation of learning model that not all college-aged learners constructively apply metacognitive strategies to maximize their learning while conserving their resources (Dunlosky et al., 2005; Zimmerman & Schunk, 2001). The suggestion for educators, therefore, is that they must recognize that some students may work against their own self-interests in attempting to solve some problems in vain and therefore may require explicit coaching on how to make their learning effective and efficient (e.g., Walberg, 1982).

One may argue, however, that unsolvable problems are rarely present in educational settings and perhaps are even unfair to pose to students. We argue otherwise given that the goal of education is to prepare students to learn outside of the classroom and under natural ecological conditions where problems frequently do not have a criterion controlled by some external force and do not have an answer that is objectively correct. Using questions and activities based in divergent-thinking (such as problem-based, project-based, or case-studies) encourages searching for a range of answers and therefore having instructors allocate more mental and temporal resources in classes to these types of problems allows for all options to be analyzed, thus optimizing what an appropriate solution would look like.

### 4.4. Limitations

The present study contains some limitations that we must address. First, an inductive reinforcement-learning problem involving perceptual discriminations was used. Although feedback-based RL is a ubiquitous learning process, we cannot affirm that the performance and individual differences observed in the present study would generalize to other tasks. Further, we acknowledge that our task was an experimentally derived analogue of open-ended learning problems conducted under controlled laboratory conditions. The generalization of these results should be explored with another version of the task that allows for the manipulation of problem outcome. Replication under ecological classroom conditions is also warranted.

A second shortcoming is shared with other studies using college students is that the sample did not include a diverse array of demographics, as convenience sampling was used in which participants could choose to participate. Since random sampling techniques were not used, the sample is likely not representative of age, gender, or race as most of the sample was male and Caucasian. We may speculate from research on self-regulated learning that suggests female students may show stronger self-monitoring and time-management strategies than male students (Liu et al., 2021), which might predict more efficient cessation on the solvable and overlearning problems. For racial and ethnic minority students, the stereotype threat literature suggests that concerns about confirming negative group stereotypes can either prompt premature disengagement or drive excessive persistence as compensatory effort (Beasley & Fischer, 2012; Steele & Aronson, 1995). Because the present sample cannot test between these possibilities, future research should recruit a more demographically representative sample and directly test whether gender and race/ethnicity moderate the relation between learning and cessation behavior and academic outcomes.

The third limitation is that although the sample was robust enough to ensure necessary statistical power, the amount of variance accounted for in the regression was very low even though significant effects were found. There are several unaccounted-for reasons that could influence academic outcomes that were not entered as predictors. The fact that the effects of cessation were still able to be teased out speaks to their relative importance.

Finally, we must address the use of 0.30 as the criterion for curve stabilization. We openly acknowledge that this cutoff is not justified by precedent in the literature, as none exists for this specific measure. Ours was a novel methodological approach that could not rely on prior usage. The choice was not arbitrary, however: sensitivity analyses were conducted to examine how stabilization points shifted under alternative criteria. A higher criterion (0.40) yielded earlier stabilization points, whereas a lower criterion (0.20) produced stabilization points that were considerably later. Using a 0.30 change in error rate from the previous trial represented an intermediate choice that avoided both extremes. Further, selecting a criterion point to define change in performance without strong prior usage has some precedent in other areas of human behavioral research. Habituation research with infants, for example, involves a comparable process of decrement in a repeated-trial response and conventionally relies on a criterion of 50% decline from initial looking time to define stabilization (Colombo & Mitchell, 2009; Dannemiller, 1984; Horowitz et al., 1972). Similarly, single-case research design methodology defines phase stability using a criterion in which a specified percentage of data points must fall within a set band around the mean (Kratochwill et al., 2013). These examples establish a broader precedent for defining stabilization through an a priori percentage-based cutoff. Given the novelty of both the approach and the criterion used here, parametric studies systematically examining how criterion selection affects outcome measures would be a valuable direction for future studies.

### 4.5. Future Directions

In addition to studies that address the limitations noted above, some plausible hypotheses regarding why some learners failed to be responsive to their on-going learning performance will be explored in future investigations.

First, there may be a problem detecting and monitoring the current state of performance. In other words, learners may not be sensitive to the feedback for their performance; this is supported by the metacognitive illusion’s assertion that learners may estimate they are doing better than they actually are (Cervin-Ellqvist et al., 2020). Further, there are findings that humans have difficulty learning from failures or past errors (Eskreis-Winkler & Fishbach, 2019, 2022), even though there is educational benefit to be gained from making and correcting errors (Metcalfe, 2017).

A second potential set of reasons why some individuals cannot cease learning may be related to temperamental or personalistic factors (Evans & Rothbart, 2007; Rothbart et al., 2007) such as having a goal versus performance orientation (Elliott & Dweck, 1988; Wang et al., 2021) and, of course, whether grit plays a role in cessation (Duckworth et al., 2012a, 2012b).

## 5. Conclusions

This study demonstrates that college learners showed wide variations in self-regulation: they continued attempting problems well past the point where further effort was useful, whether they had already mastered the task or were facing an unsolvable one. Importantly, individual differences in cessation behavior predicted academic outcomes. Faster disengagement from a completed (solvable) task was linked to higher ACT scores and high school GPA, while greater persistence on the unsolvable problem was also associated with better ACT and university GPA performance. These conclusions are nuanced by the type of problem and outcome measured. These findings introduce important qualification to understanding the self-regulation of learning by showing that a utility-driven approach to learning is not universally applied by all students.

## Figures and Tables

**Table 1:** Trials in Each Phase of the Learning Curve.

Problem	Phase	Mean	*SD*	Median
Solvable	Learning (CritTrial)	11.75	7.49	10
	Cessation (TrialsToEnd)	8.49	14.9	3
Overlearning	Learning (CritTrial)	36.96	19.76	41.5
	Cessation (TrialsToEnd)	10.15	13.28	6
Unsolvable	Learning (CritTrial)	13.65	13.78	9
	Cessation (TrialsToEnd)	40.39	18.65	47

**Table 2:** Predicting Academic Outcome from Number of Trials.

Outcome	Phase	Problem	Beta Coefficient	*t*	*p*
ACT	Learning	Solvable	−0.127	−1.532	0.128
		Overlearning	−0.221	−2.381	0.019 *
		Unsolvable	0.129	1.262	0.209
	Cessation	Solvable	−0.220	−2.590	0.011 *
		Overlearning	−0.099	−1.033	0.303
		Unsolvable	0.310	2.907	0.004 *
University GPA	Learning	Solvable	−0.115	−1.248	0.214
		Overlearning	−0.079	−0.801	0.425
		Unsolvable	0.013	0.117	0.907
	Cessation	Solvable	−0.111	−1.235	0.219
		Overlearning	0.122	1.191	0.236
		Unsolvable	0.231	1.961	0.052 +
HS GPA	Learning	Solvable	−0.181	−2.235	0.027 *
		Overlearning	0.001	0.013	0.990
		Unsolvable	−0.051	−0.509	0.612
	Cessation	Solvable	−0.219	−2.675	0.008 *
		Overlearning	0.171	1.853	0.066 +
		Unsolvable	0.053	0.499	0.618

* Refers to significance at the 0.05 level or less; + refers to marginal significance.

## Data Availability

The data and analyses are available at Coldren, J. (2026). *Individual differences in learning and cessation predict academic performance* [Data set]. OSF. https://osf.io/fjqvz (accessed on 12 August 2026) (Coldren, 2026).
